# Safety of EVO ICL Implantation With an Ophthalmic Viscosurgical Device-Free Technique in the Early 24 h After Surgery

**DOI:** 10.3389/fmed.2021.764653

**Published:** 2021-11-17

**Authors:** Zhe Zhang, Lingling Niu, Jing Zhao, Huamao Miao, Zhuoyi Chen, Yang Shen, Xun Chen, Yuhao Ye, Xiaoying Wang, Xingtao Zhou

**Affiliations:** ^1^Department of Ophthalmology, Eye Institute, Eye and ENT Hospital, Fudan University, Shanghai, China; ^2^NHC Key Laboratory of Myopia, Fudan University, Shanghai, China; ^3^Key Laboratory of Myopia, Chinese Academy of Medical Sciences, Shanghai, China; ^4^Shanghai Research Center of Ophthalmology and Optometry, Shanghai, China; ^5^Shanghai Engineering Research Center of Laser and Autostereoscopic 3D for Vision Care, Shanghai, China

**Keywords:** ophthalmic viscosurgical device (OVD), intraocular pressure (IOP), implantable collamer lens (ICL), endothelial cell density (ECD), corneal densitometry

## Abstract

**Purpose:** To compare the safety of the non-ophthalmic viscosurgical device (OVD) technique with that of the minimum OVD technique in EVO Implantable Collamer Lens (EVO-ICL) implantation.

**Methods:** A total of 180 eyes of 90 consecutive patients were enrolled in the study, of which 100 eyes of 50 patients were treated with non-OVD technique, with a 55% success rate. The remaining 80 eyes of 40 patients were treated with min-OVD technique, so they were classified into the min-OVD group. Preoperative and postoperative intraocular pressure (IOP) measurements were collected and analyzed at 1, 2, 3, and 24 h. Visual acuity, corneal endothelial cell density (ECD), and corneal densitometry 24 h postoperatively were evaluated.

**Results:** No significant difference was found in visual outcomes (*P* = 0.54) or ECD (*P* = 0.78) between the two groups. The operation time was significantly shorter in the non-OVD group (*P* < 0.0001). The IOP was significantly higher at 1 h (*P* < 0.0001), 2 h (*P* < 0.0001) and 3 h (*P* = 0.0045) postoperatively in the min-OVD group. The non-OVD group had significantly lower IOP than the min-OVD group at 1 h (*P* = 0.01) and 2 h (*P* = 0.013) postoperatively. The temporal corneal densitometry in the non-OVD group were significantly lower than those in the minimum group (*P* = 0.0063) 1 day after surgery.

**Conclusion:** The non-OVD technique is safe and efficient for ICL implantation. It can be a safer method of ICL implantation in that it completely eliminates ophthalmic viscoelastic devices related complications without causing additional complications in short term.

## Introduction

Myopia has become a worldwide public health issue (1, 2). Visian Implantable Collamer Lens (ICL) implantation has been reported to be a safe and effective way to correct myopia (3–6). Ophthalmic viscosurgical device (OVD) for ICL implantation is widely used to protect endothelial cells and maintain anterior chamber stability during surgery (7). Retained OVDs are the major cause of early (within 24 h postoperatively) acute IOP elevation (8–13). It has become well accepted that retained viscoelastic materials mechanically obstructs the trabecular outflow pathway and decreases the outflow facility (14–16), which is the main reason for the early postoperative intraocular pressure (IOP) elevation. IOP spikes of 30 mmHg or higher in the early period after surgery may be associated with corneal epithelial edema and pain and may increase the risk of retinal artery occlusion and anterior ischemic optic neuropathy (17–19). To prevent a postoperative IOP increase, complete removal of the OVD is of high importance.

The min-OVD technique has been applied by experienced surgeons in recent years (20, 21). The application of this technique can greatly reduce the occurrence of high IOP after surgery, and postoperative recovery is rapid. On the basis of the one-step min-OVD technique, we further invented the non-OVD technique. It can completely eliminate the use of the OVD and balanced salt solution (BSS) under the premise of maintaining the stability of the anterior chamber during the operation. With this technique, the risk of interference to the eyes during the operation will be minimal.

Due to the rapid postoperative recovery of the non-OVD technique, it is necessary to observe the early postoperative outcomes. Therefore, we designed a prospective study to observe the outcomes of ICL implantation without the use of OVD 1, 2, 3, and 24 h after surgery. The purpose of this prospective study was to evaluate the safety and efficacy of ICL implantation without the use of OVD.

## Methods

### Study Design

This study was conducted at the Eye and ENT Hospital of Fudan University (Shanghai, China). All patients were fully informed of the details and potential risks of the procedure, and written informed consent was obtained from all patients. This study was approved by the Ethical Committee of the Eye and ENT Hospital of Fudan University, and all work was carried out in accordance with the Declaration of Helsinki.

A total of 90 continuous subjects (180 eyes) were recruited from August 2020 to October 2020 to participate in this study for applying non-OVD technique. If the patient was not successfully performed with OVD technique during surgery, we will apply min-OVD technique for patients and act as control group.

Participants were aged between 20 and 40 years, had stable refractive error (≤0.5 D change in refractive error in the past 2 years) and did not use contact lenses for 2 weeks. Exclusion criteria comprised eye disorders or systemic disease, IOP < 21 mmHg, anterior chamber depth (ACD) <2.8 mm and corneal endothelial cell density (ECD) <2,000 cells/mm^2^.

### Examination

All patients underwent a standard ocular examination, including uncorrected distance visual acuity (UDVA), corrected distance visual acuity (CDVA), manifest refraction, slit lamp examination, corneal topography (Pentacam HR, Oculus Optikgeräte GmbH, Wetzlar, Germany), ACD (Oculus, Wetzlar, Germany), axial length (IOLMaster, Zeiss Humphrey, Carl Zeiss Meditec, Inc., Dublin, CA) and IOP (Canon, Japan). The ICL (EVO Visian ICL; STAAR Surgical AG) power was chosen using a modified vertex formula according to the manufacturer's suggestion. Lens size was calculated through the same protocol based on the white-to-white ratio and ACD. Data for UDVA, CDVA, the corneal densitometry value of the whole cornea and ECD were collected at 24 h postoperatively. IOP was monitored 1, 2, 3, and 24 h postoperatively.

### Surgical Procedure

All surgeries were performed by the same surgeon (XZ) using surface anesthesia with 0.4% oxybuprocaine. In the non-OVD group, no OVD or BSS was injected into the anterior chamber during the whole surgical procedure. ICL was implanted via a 3-mm temporal corneal incision. The anterior chamber depth is stable, indicating that a good self-closing incision has been made. ICL lens is implanted by an injector cartridge and unfolds smoothly, then, two distal haptics slowly reach the direction of the ciliary sulcus, then slowly push ICL, and gently press the two proximal haptics, and push the two proximal haptics under the iris. During the injection of ICL, the four haptics were directly implanted into the posterior chamber in one step, or all three haptics were implanted into the posterior chamber, and the proximal one haptic was not implanted into the posterior chamber. The min-OVD technique applied in the situation of one or two ICL haptics were inserted into the posterior chamber at one time. In the min-OVD group, the same procedure implemented as that in the non-OVD group at the beginning. After the ICL was inserted, a minimal amount of OVD was injected in front of the ICL to slightly adjust its position, and then a BSS was used to wash the OVD out of the anterior chamber (20, 22). The differences between the two methods are listed in Table 1. The operation time was calculated for each surgery (the time calculated from making the first corneal incision to closing the incision at the end). After the surgery, a topical antibiotic (0.5% levofloxacin, Cravit, Santen, Osaka, Japan) was administered four times per day for 7 days. A topical steroid (1.0% prednisolone acetate, Pred Forte; Allergan, Irvine, CA, USA) was used four times daily for 4 days, pranoprofen (Senju, Osaka, Japan) was used four times daily for 14 days and Natriumhyaluronat (Hycosan, Germany) was used four times daily for 3 months.

**Table 1:** Comparison of the two methods of ICL implantation.

	**Non-OVD group** **(*n* = 100)**	**Min-OVD group** **(*n* = 80)**
Number of haptics inserted at one time	3 or 4	1 or 2
OVD usage	No	Yes
OVD removal	No	Yes
BSS usage	No	Yes

### Corneal Densitometry

Corneal densitometry was measured from Scheimpflug images and was expressed in gray scale. The mean densitometry values on the diameters from 0–2 mm to 2–6 mm of the corneas' anterior layer (the first 120 μm of the complete corneal thickness), posterior layer (the last 60 μm of the complete corneal thickness) and central layer (the volume between the anterior layer and the posterior layer), the nasal and temporal total layer (the volume between the epithelium and endothelium of a cornea) were analyzed.

### Statistical Analyses

All statistical analyses were performed using SPSS version 23 (IBM Corp, USA). The data were presented as the mean ± standard deviation. Normality of data was assessed using the Shapiro-Wilk test, and data were normal in all cases. The baseline variables, were compared using Student's *t*-test. Comparison of sex was conducted using the chi-square test.

The IOP, ECD and corneal densitometry were also compared using repeated-measures ANOVA, with time being the intragroup factor and treatment modality being the intergroup factor. Tukey's multiple comparison tests were used for pairwise comparisons. A *P* < 0.05 was considered statistically significant.

## Results

### Subjects and Baseline Biometrics

According to the surgical procedures during the operation and the repeated watching of the surgical video postoperatively, we statistically concluded that the success rate of non-OVD technique in these patients was 55.6%. 100 eyes of 50 patients were treated with non-OVD technique. Eighty eyes of 40 patients were unsuccessful with non-OVD technique and were treated with the traditional surgery method (min-OVD technique). Baseline biometrics and comparisons among groups are shown in Table 2. No significant differences were found between the two groups in baseline biometrics. All surgeries were uneventful, and no intraoperative complications were observed. No postoperative adverse events were recorded in either group, except for the expected changes in IOP.

**Table 2:** Biometric data of subjects at baseline.

**Characteristic**	**Non-OVD group** **(*n* = 100)**	**Min-OVD group** **(*n* = 80)**	***P*-value**
Sex (M/F)	16/34	13/27	0.50
Age (years)	26.27 ± 5.23	25.33 ± 5.98	0.25
Axial length (mm)	26.95 ± 4.19	27.05 ± 1.61	0.86
UDVA (logMAR)	0.07 ± 0.06	0.08 ± 0.04	0.67
CDVA (logMAR)	0.98 ± 0.20	1.02 ± 0.10	0.29
SE (D)	−6.19 ± 5.33	−6.82 ± 4.76	0.42
IOP (mmHg)	15.48 ± 2.68	15.98 ± 3.40	0.27
ACD (mm)	3.21 ± 0.25	3.20 ± 0.29	0.81
ICL size (mm)	12.62 ± 0.35	12.58 ± 0.35	0.73

### Efficacy and Safety

**Figure 1:**
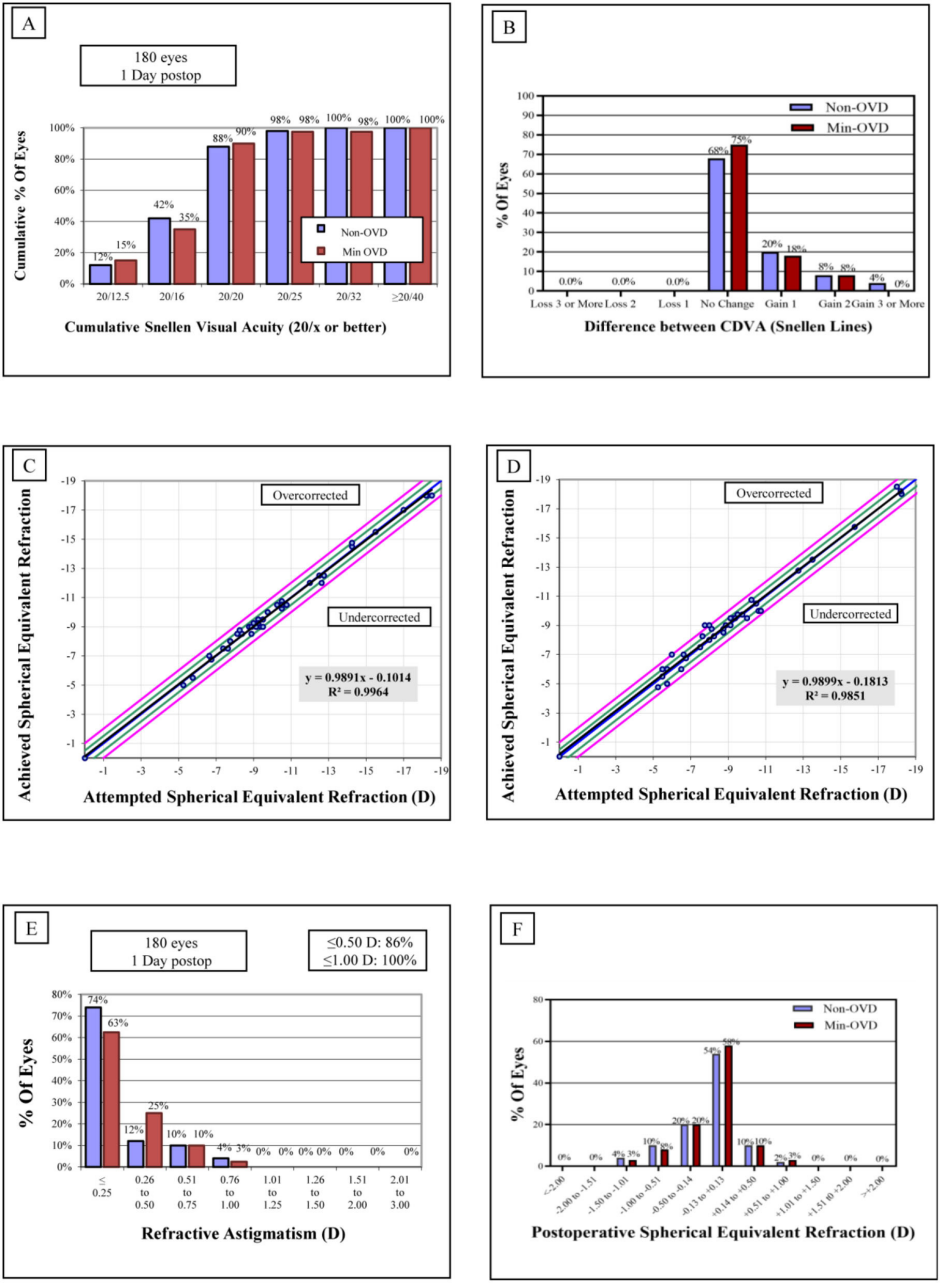
Refractive outcomes 1 day after implantable collamer lens (ICL). **(A)** Cumulative uncorrected distance visual acuity (UDVA). **(B)** Changes of corrected distance visual acuity (CDVA). **(C,D)** Attempted vs. achieved SE refraction after ICL in the non-OVD group **(C)** and min-OVD group **(D)**. **(E)** Refractive astigmatism. **(F)** Spherical equivalent (SE) refraction.

The mean efficacy indices (postoperative UDVA/preoperative CDVA) were 1.05 ± 0.15 and 1.08 ± 0.17 in the non-OVD group and min-OVD group, respectively (*P* = 0.52). The safety indices (postoperative CDVA/preoperative CDVA) were 1.15 ± 0.19 and 1.22 ± 0.23 in the non-OVD group and min-OVD group, respectively (*P* = 0.24). No eyes in either group lost one or more lines of CDVA. Figure 1 shows the visual outcomes of the two groups 1 day after surgery. The vault of non-OVD group and min-OVD group was 553.8 ± 195.9 μm and 511.4 ± 147 μm, respectively (*P* = 0.28).

### Operation Time

In the non-OVD group, the operation time was 63.68 ± 27.35 s. The distribution of the operation time was as follows: 60 eyes (60%) took <1 mine and 40 eyes (40.0%) took <2 min. In the min-OVD group, the operation time was 144.8±33.27 s. The distribution of the operation time was as follows: 28 eyes (35%) took <1 min, 40 eyes (50.0%) took <2 min, and 12 eyes (15%) took <3 min. The operation time was significantly shorter in the non-OVD group (*P* < 0.0001).

### Intraocular Pressure

**Figure 2:**
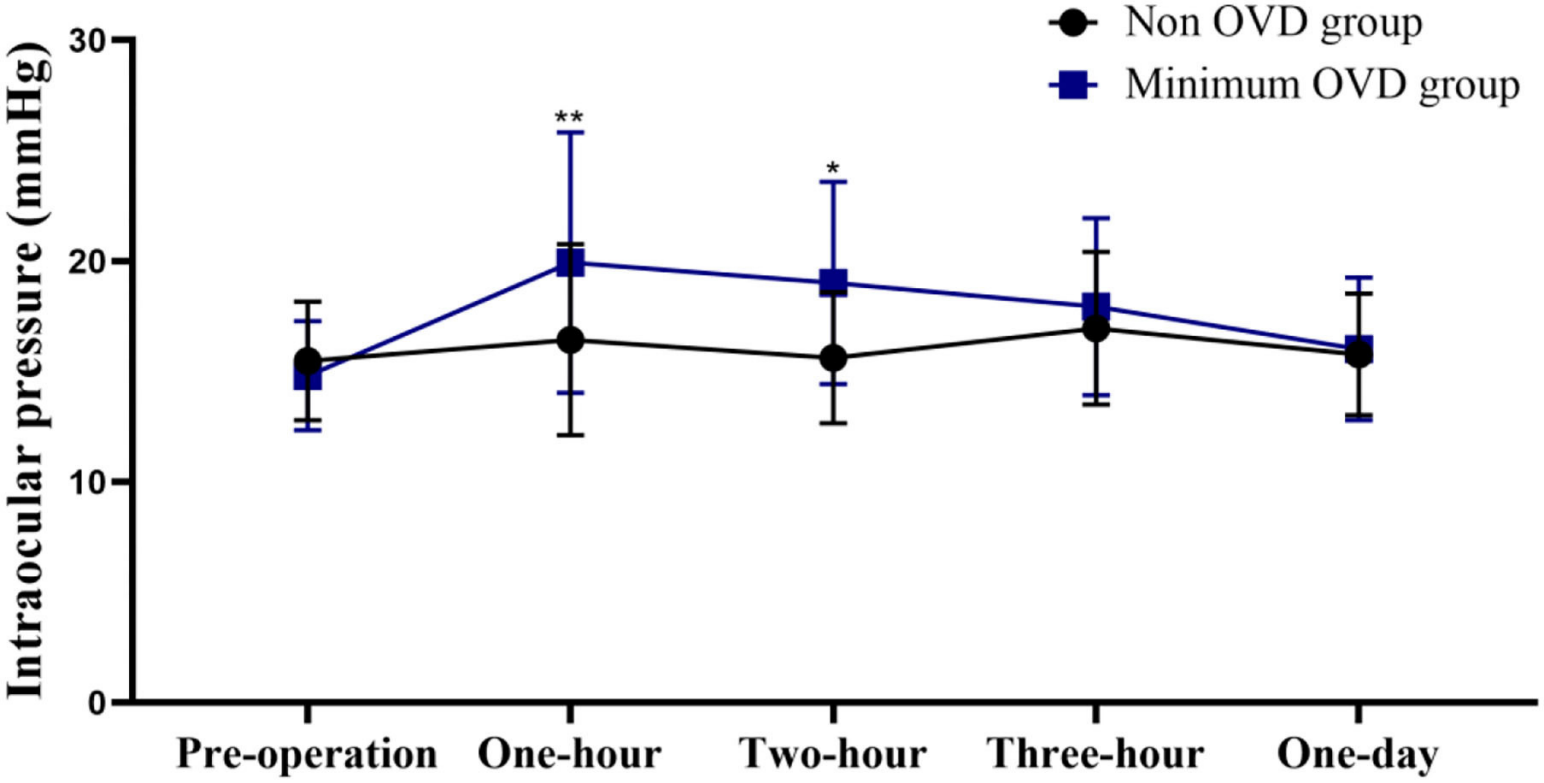
Intraocular pressure (IOP) of the non-OVD group and min-OVD group at each follow-up time point. Error bars represent standard deviation, *P < 0.05; **P < 0.01.

The postoperative IOP was more stable in the non-OVD group than in the min-OVD group. The IOP was significantly higher than the preoperative value at 1 h (*P* < 0.0001), 2 h (*P* < 0.0001), and 3 h (*P* = 0.0045) postoperatively in the min-OVD group, while in the non-OVD group, there was no significant difference between preoperative intraocular pressure and postoperative intraocular pressure at each time point. The non-OVD group had significantly lower IOP than the min-OVD group at 1 h (*P* = 0.01) and 2 h (*P* = 0.013) postoperatively. There was no differences were found between the two groups after 24 h. The occurrence rate of paracentesis tap was significantly lower in the non-OVD group than in the min-OVD group (1% [1 of 100] vs. 5% [4 of 80], *P* < 0.01). Moreover, the use of IOP control drugs was significantly lower in the non-OVD group than in the min-OVD group (0 [0 of 100] vs. 3.8% [3 of 80], *P* < 0.01) (Figure 2).

### Endothelial Cell Density

There was no significant difference between groups at each follow-up time point. In the min-OVD group, the ECD changed from 2,627 ± 202 cells/mm^2^ preoperatively to 2,571 ± 241 cells/mm^2^ at 3 h and to 2,586 ± 280 cells/mm^2^ 1 day postoperatively (*P* > 0.05). In the non-OVD group, the ECD changed from 2,645 ± 204 cells/mm^2^ preoperatively to 2,596 ± 255 cells/mm^2^ at 3 h and to 2,619 ±182 cells/mm^2^ 1 day postoperatively (*P* > 0.05). In addition, there was no significant difference in the coefficient of variation (CV) or hexagonal cell ratio (6A) between groups at each follow-up time point. In the min-OVD group, the CV changed from 42.04 ± 8.40 preoperatively to 40.06 ± 7.14 at 3 h and to 41.15±5.17 1 day postoperatively (*P* > 0.05). In the non-OVD group, the CV changed from 41.39 ± 5.90 preoperatively to 42.07 ± 5.95 at 3 h and to 40.91 ± 4.91 1 day postoperatively (*P* > 0.05). In the min-OVD group, the 6A changed from 40.75 ± 7.15 preoperatively to 40.12 ± 8.09 at 3 h and to 39.65 ± 6.69 1 day postoperatively (*P* > 0.05). In the non-OVD group, the 6A changed from 42.06 ± 6.86 preoperatively to 41.15 ± 9.16 at 3 h and to 40.97 ± 7.71 1 day postoperatively (*P* > 0.05).

### Corneal Densitometry

**Figure 3:**
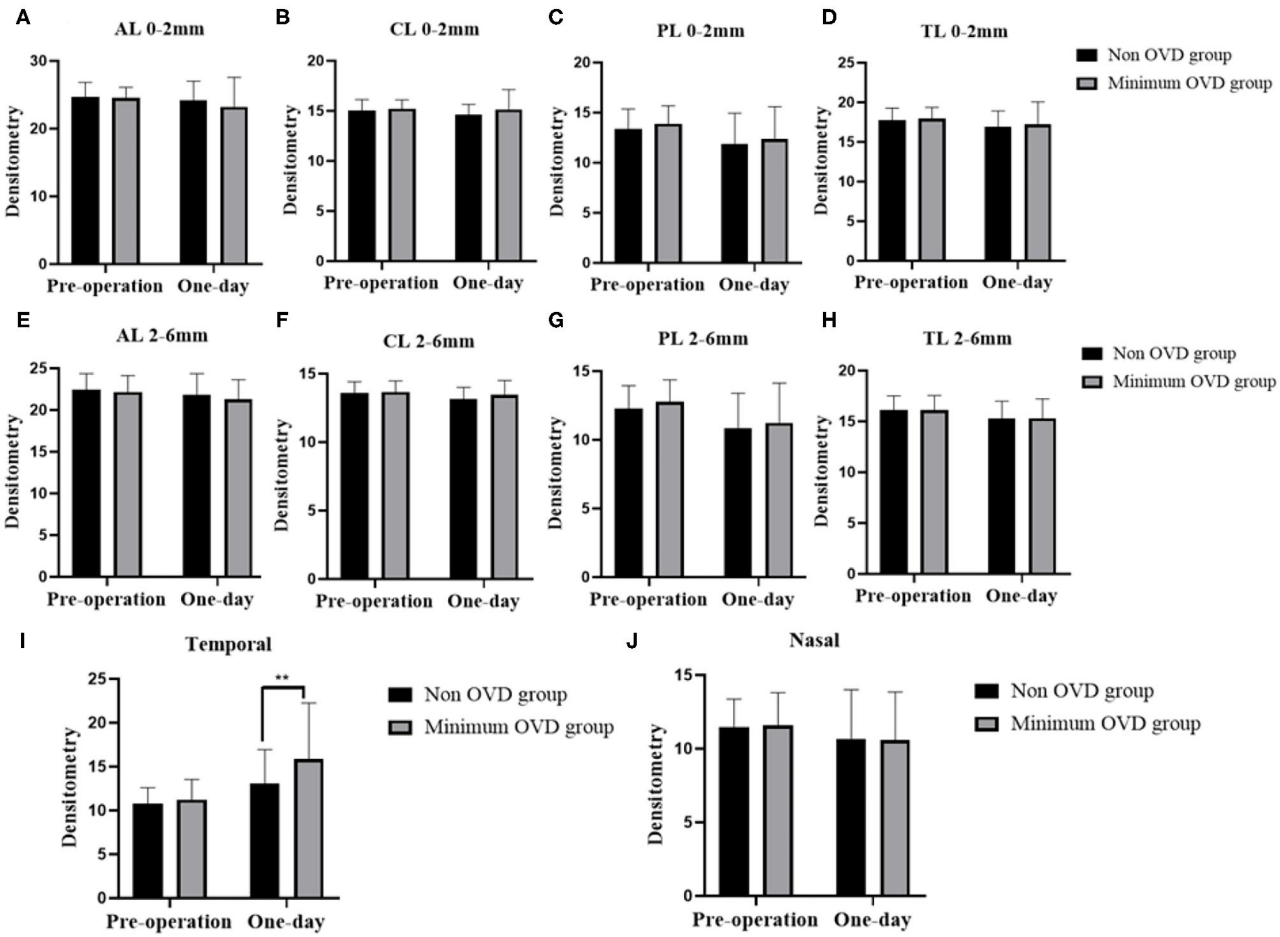
Densitometry of the cornea in the non-OVD group and min-OVD group at different time points postoperatively. Corneal densitometry values of the anterior layer **(A)**, central layer **(B)**, posterior layer **(C)** and total layer **(D)** from 0 to 2 mm. Corneal densitometry values of the anterior layer **(E)**, central layer **(F)**, posterior layer **(G)** and total layer **(H)** from 2 to 6 mm. Corneal densitometry values of temporal **(I)** and nosal **(J)** part. **P < 0.01, the error bar indicates the standard deviation.

Regarding the annular diameters of 0–2 and 2–6 mm, no significant changes were detected in the corneal densitometry values of the anterior layer (AL 0–2 mm and 2–6 mm), the central layer (CL 0–2 mm and 2–6 mm), the posterior layer (PL 0–2 mm and 2–6 mm) the total layer (TL 0–2 mm and 2–6 mm) or the nasal part within the first day between two groups. Temporal densitometry values assessed 1 day after the operation were significantly different between the two groups (*P* = 0.0063) and were significantly increased compared with the value obtained 1 day postoperatively (*P* < 0.0001) (Figure 3).

## Discussion

In the current study, we developed a new technique, the non-OVD technique, for use in ICL implantation. The ICL was implanted into the posterior chamber with only one corneal incision, and no OVD or BSS was injected into the anterior chamber during the whole surgical procedure. Several min-OVD techniques have been reported to implant ICLs effectively and mastered by many surgeons, it has become a common surgical method for ICL implantation. Chen et al. inserted an ICL by making a two-step corneal incision and used BSS instead of OVD. In addition, OVD is still needed when adjusting the ICL position (21). Peng et al. (23) described a technique using BSS to load the ICL and maintain the anterior chamber. Therefore, we took min-OVD technology as the control group, compared our innovative non-OVD technique with it. Compared to previous studies, our technique resulted in less intraocular interference and fewer postoperative reactions.

In our study, IOP in the min-OVD group increased significantly within 3 h postoperatively and was significantly higher than that in the non-OVD group in the first 2 h after surgery. The IOP gradually decreased to the preoperative level, and no differences were found between the two groups after 24 h. The occurrence rate of paracentesis tap and IOP control drug usage were significantly lower in the non-OVD group. Thorough removal of viscoelastic substances is vital for avoidance of a postoperative IOP increase. However, complete removal of the OVD behind the IOL is known to be difficult. It has not been achieved with any technique. Therefore, it is beneficial to maintain intraocular pressure by not using OVD during surgery.

In traditional techniques, OVD is used to protect endothelial cells and maintain anterior chamber stability during surgery (24, 25). Therefore, we paid more attention to the effect of our new technique on the morphology and number of endothelial cells in our study. Our study revealed that the non-OVD technique did not cause any additional damage to endothelial cells. No significant differences were detected between the two groups in ECD during the first 24 h postoperatively in this study. A previous study also compared the changes in endothelial cells after ICL implantation or cataract surgery with and without OVD during a long follow-up period. The results consistently showed no significant reduction in endothelial cells at the 1-year follow-up (25, 26).

Specular microscopy was used to evaluate ECD by analyzing the central corneal image, which was taken through a central fixation area of the cornea; using this technique, peripheral endothelial damage may not be detected. Corneal densitometry assessments using a Pentacam HR rather than a specular microscope are able to detect various abnormalities in a wider zone (27–30). Corneal densitometry analysis may be employed to assess endothelial function in peripheral zones. The results of the current study demonstrated that the densitometry values of AL 0–2 mm, CL 0–2 mm, PL 0–2 mm, and TL 0–2 mm remained unchanged, which was consistent with the ECD results.

Our team's previous results showed that the values of AL 2–6 mm, CL 2–6 mm, PL 2–6 mm, and TL 2–6 mm measured at 1 day postoperatively, were unchanged compared with those measured before the surgery, indicating that the effects of ICL implantation on corneal densitometry may be insignificant and that the corneal histological structure is intact. However, the results of this study showed that temporal densitometry value (corneal incision location) was significantly different between the two groups 1 day after the operation. The densitometry values of the min-OVD group increased markedly. We hypothesize that this is due to the relatively long operation time and the injection of OVD and BSS through this area, which might affect corneal densitometry (31).

The present study also suggested that the non-OVD technique could reduce surgery duration, which might make the patient feel more comfortable during the surgery and improve patient satisfaction. Compared with the traditional technique, the non-OVD technique does not require time to inject and remove the OVD and reduces the cost of consumable material and the risk of intraoperative complications. Moreover, the non-OVD technique can increase the efficiency of the surgery, which is critical for carrying out this surgery extensively and serving more patients.

The key point of the non-OVD technique is that its effectiveness is based on good self-close corneal incision making, which ensures a stable anterior chamber depth. What's more, the ICL injection is another important part of the procedure because the lens has to be kept wet. In addition, the lens incarceration in the corneal incision and rotation in the anterior chamber should be avoided. When using the non-OVD technique, if any situation emerges that impacts the anterior chamber stability, surgeons should switch to the conventional method to avoid mechanical disturbance to the corneal endothelial cell, crystalline lens, or iris.

However, the limitation of this study was that the non-OVD technique requires a longer learning curve. The non-OVD technique is best learned after traditional surgical techniques are mastered, and the promotion of non-OVD technique requires further training of ophthalmologists. The second limitation was that the non-OVD technique effect on ocular biometrics should further monitor. We originally designed a long-term study to observe the changes of these indexes.

In conclusion, the non-OVD technique is safe and efficient for ICL implantation. It can be a safer method of ICL implantation in that it completely eliminates ophthalmic viscoelastic devices related complications without causing additional complications in short term.

## Data Availability Statement

The raw data supporting the conclusions of this article will be made available by the authors, without undue reservation.

## Ethics Statement

The studies involving human participants were reviewed and approved by Ethical Committee of the Eye and ENT Hospital of Fudan University. The patients/participants provided their written informed consent to participate in this study.

## Author Contributions

XZ and XW designed and directed the project. ZZ, LN, XC, YY, and ZC performed the experiments. YS and HM analyzed spectra. ZZ and LN wrote the article. All authors contributed to the article and approved the submitted version.

## Funding

This work was supported by the National Natural Science Foundation of China (81770955), Joint Research Project of New Frontier Technology in Municipal Hospitals (SHDC12018103), and Project of Shanghai Science and Technology (grant no. 20410710100).

## Conflict of Interest

The authors declare that the research was conducted in the absence of any commercial or financial relationships that could be construed as a potential conflict of interest.

## Publisher's Note

All claims expressed in this article are solely those of the authors and do not necessarily represent those of their affiliated organizations, or those of the publisher, the editors and the reviewers. Any product that may be evaluated in this article, or claim that may be made by its manufacturer, is not guaranteed or endorsed by the publisher.
